# Long‐Term Safety of Intradermal Polynucleotides in the Periocular Region: A Retrospective Study

**DOI:** 10.1111/jocd.71029

**Published:** 2026-07-01

**Authors:** Elena Caride Miana

**Affiliations:** ^1^ Clinica Dra Caride Alicante Spain; ^2^ Universidad Miguel Hernadez de Elche Alicante Spain

**Keywords:** adverse events, aesthetic medicine, periocular rejuvenation, polynucleotides, real‐world evidence, safety

## Abstract

**Background:**

The use of polynucleotides (PN) has increased significantly in aesthetic medicine due to their regenerative properties and favorable short‐term safety profile. However, evidence regarding their long‐term safety, particularly in real‐world clinical practice, remains limited.

**Objective:**

To evaluate the short‐ and long‐term safety of intradermal polynucleotide treatment (PN‐HPT) in the periocular region based on routine clinical practice.

**Methods:**

A retrospective observational study was conducted through medical record review. Patients consecutively treated with PN‐HPT for periocular rejuvenation between January and March 2024 were included. This inclusion period was defined to ensure a minimum available timeframe of 24 months from the first treatment session at the time of data analysis. All patients completed an initial protocol of three sessions, administered at 4‐week intervals. Polynucleotides (PN‐HPT 7.5 mg/mL) were administered intradermally using a 33G needle, with 1 mL injected per periocular side. Data on treatment sessions and adverse events were collected from clinical records. As part of routine clinical care, all patients were systematically contacted 24 h after each treatment session to assess early adverse events, and underwent at least one in‐person evaluation approximately 1 month after the final session. Subsequently, clinical records of all included patients were reviewed up to March 2026 to identify any additional or delayed adverse events. Safety assessment was based on documented events during routine clinical practice.

**Results:**

A total of 23 patients were included, accounting for 91 treatment sessions between January 2024 and March 2026. Fifteen patients (65.2%) underwent additional sessions beyond the initial protocol, reflecting repeated exposure under real‐world conditions. Only two mild and self‐limited adverse events were documented: one case of transient periocular edema and one case of prolonged intradermal papules. No moderate or severe adverse events were recorded. Importantly, no delayed adverse events were identified in the medical records during the study timeframe. All patients continued receiving care during this period, and no additional treatment‐related adverse events were documented.

**Conclusions:**

In this retrospective real‐world study, PN‐HPT demonstrated a favorable safety profile in the periocular region, with minimal and transient adverse events and no evidence of delayed complications despite repeated treatments. These findings support the tolerability of polynucleotides in routine clinical practice. Further prospective studies with larger sample sizes and standardized follow‐up are warranted to confirm long‐term safety.

## 1 Introduction

The use of polynucleotides (PN) has expanded substantially in aesthetic and regenerative medicine over the past decade, driven by the growing demand for minimally invasive treatments that not only improve skin quality but also promote tissue regeneration with a favorable safety profile. Polynucleotides are highly purified DNA fragments, typically derived from salmonid species, which undergo extensive processing to enhance biocompatibility and minimize immunogenic potential [1, 2].

Unlike traditional injectable treatments that primarily provide volumization or structural support, PN are considered part of the regenerative medicine paradigm due to their capacity to modulate tissue repair processes. Experimental and clinical evidence suggests that PN can stimulate dermal fibroblast activity, promoting the synthesis of extracellular matrix components, including collagen, elastin, and glycosaminoglycans while also exerting anti‐inflammatory and tissue‐protective effects [3, 4]. These properties have contributed to their increasing use in aesthetic practice, particularly for skin rejuvenation, improvement of dermal quality, and treatment of delicate anatomical areas such as the periocular region.

Despite their widespread clinical adoption, robust evidence regarding the long‐term safety of PN remains limited. Most available studies have focused on short‐term outcomes, primarily addressing efficacy and immediate tolerability, often within controlled or experimental settings and with relatively short follow‐up periods [5]. As a result, there is a notable lack of data on delayed or late‐onset adverse events, particularly in the context of repeated treatments and real‐world clinical use [6].

This gap is especially relevant in aesthetic medicine, where cumulative exposure to injectable treatments and their long‐term safety are of increasing concern. Real‐world evidence derived from routine clinical practice may therefore provide valuable complementary insights into the safety profile of PN beyond controlled study conditions.

In this context, the present study aims to evaluate the occurrence of adverse events associated with intradermal PN treatment in the periocular region, based on retrospective analysis of routine clinical data over an extended timeframe. By focusing on real‐world practice and cumulative treatment exposure, this study seeks to contribute to the limited evidence available on the long‐term safety of polynucleotides in aesthetic medicine.

## 2 Methods

A retrospective observational study was conducted based on the review of medical records from routine clinical practice in an aesthetic setting. Consecutive patients treated with polynucleotides (PN‐HPT 7.5 mg/mL) for periocular rejuvenation between January and March 2024 were included. This inclusion window was intentionally defined to ensure that, at the time of data extraction, all patients had a minimum available timeframe of 24 months from the first treatment session, enabling the assessment of potential delayed adverse events.

All included patients completed a standardized initial treatment protocol consisting of three sessions. Polynucleotides were administered intradermally in the periocular region, with 1 mL injected per side using a 33G needle. Treatment sessions were performed at 4‐week intervals. All procedures were carried out under routine clinical conditions according to standard practice.

Data were retrospectively extracted from medical records, including patient demographics, treatment characteristics, number of sessions, and any documented adverse events. As part of routine clinical care, all patients were systematically contacted 24 h after each treatment session to assess early adverse events. In addition, all patients underwent at least one in‐person clinical evaluation approximately 1 month after completion of the initial treatment protocol.

No protocolized long‐term follow‐up was implemented for research purposes. However, all patients remained under ongoing clinical care at the center, allowing for continuous documentation of any treatment‐related events in the medical records. To assess long‐term safety, clinical records of all included patients were reviewed up to March 2026, capturing all documented adverse events within routine practice.

The primary outcome was the occurrence of treatment‐related adverse events. When applicable, adverse events were classified according to time of onset as immediate, early, or delayed. Safety outcomes were analyzed both per patient and per treatment session to account for cumulative exposure.

The aim of this study was to evaluate the short‐ and long‐term safety profile of intradermal polynucleotide treatment in the periocular region under real‐world clinical conditions.

## 3 Results

A total of 23 consecutive patients treated with PN‐HPT between January and March 2024 were identified through medical record review. All patients were female. Each patient completed the initial treatment protocol consisting of three sessions. Baseline characteristics are summarized in Table 1.

**TABLE 1:** Baseline characteristics of the study population.

Variable	Value
Number of patients	23
Sex (female), *n* (%)	23 (100%)
Initial sessions per patient	3
Total treatment sessions	91
Patients with additional sessions, *n* (%)	15 (65.2%)

Between January 2024 and March 2026, a total of 91 treatment sessions were performed across the cohort, as documented in the medical records. Of the 23 patients, 15 (65.2%) underwent additional sessions beyond the initial three‐session protocol, reflecting repeated exposure to PN‐HPT in routine clinical practice.

Overall, treatment was well tolerated; adverse events are presented in Table 2. Two mild and self‐limited adverse events were recorded, corresponding to 2/91 sessions (2.2%) and affecting 2/23 patients (8.7%). One patient (4.3%) developed transient periocular edema approximately 12 h after treatment, which resolved spontaneously without the need for medical intervention. Another patient (4.3%) presented persistence of intradermal papules at the injection site for up to 24 h, which also resolved without treatment.

**TABLE 2:** Treatment‐related adverse events.

Adverse event	Patients, *n* (%)	Sessions, *n* (%)	Description
Periocular edema	1 (4.3%)	1 (1.1%)	Self‐limited, resolved without treatment
Persistent intradermal papules	1 (4.3%)	1 (1.1%)	Resolved within 24 h
Moderate/severe adverse events	0	0	None reported
Delayed adverse events	0	0	None reported
Total adverse events	2 (8.7%)	2 (2.2%)	

No moderate or severe adverse events were recorded. Importantly, no delayed adverse events were identified in the medical records during the study timeframe.

At the 1‐month post‐treatment assessment following completion of the initial protocol, no patients reported or presented adverse effects. All patients continued receiving care at the clinic throughout the study timeframe, and no additional treatment‐related adverse events were documented in the medical records.

## 4 Discussion

One of the most relevant findings of this study is the absence of delayed adverse events documented in the medical records over an extended timeframe. While most published data on PN focus on short‐term outcomes and immediate tolerability, evidence regarding long‐term safety remains limited. In this context, our findings contribute to the growing body of literature supporting the safety of PN, particularly in the periocular area, which is considered a high‐risk anatomical region in aesthetic practice.

Importantly, the cumulative exposure observed in this cohort strengthens the interpretation of safety outcomes. Although the number of patients is limited, the total number of procedures (*n* = 91) and the fact that a substantial proportion of patients (65.2%) continued receiving treatments beyond the initial protocol; this repeated exposure, in the absence of reported adverse events, supports the tolerability of PN‐HPT under routine clinical conditions.

Regarding the adverse events observed, both were mild and self‐limited. One patient developed transient periocular edema approximately 12 h after treatment. Notably, during the same session, the patient had also received botulinum toxin in the upper facial third. It may be hypothesized that the pharmacological effect of botulinum toxin, particularly its action on the orbicularis oculi muscle, could have temporarily reduced lymphatic drainage capacity in the periocular region [7, 8]. This, in turn, may have influenced the transient accumulation of intradermally injected product (1 mL per periocular side), contributing to the development of edema. Although causality cannot be established, this observation highlights a potential interaction between treatments and suggests that combined procedures in the periocular area should be approached with consideration of their physiological effects.

The second adverse event consisted of prolonged persistence of intradermal papules for up to 24 h following injection. Given that PN‐HPT is administered intradermally, the formation of transient papules is an expected and well‐known phenomenon associated with this technique. Variability in skin quality and dermal characteristics among patients may influence the duration of papule resolution. In this case, the event was self‐limited and resolved without intervention, supporting its benign and procedure‐related nature.

No patients reported or presented long‐term adverse events during the study timeframe. Additionally, no patients were lost to follow‐up within the context of routine clinical care, and all continued attending the clinic during the study period.

This study has several limitations that should be acknowledged. First, its retrospective design inherently relies on the accuracy and completeness of medical records, which may lead to underreporting of mild or transient adverse events, particularly if patients did not seek medical attention. Second, although all patients underwent a clinical evaluation 1 month after the final treatment session and continued receiving care at the center during the study timeframe, no protocolized follow‐up specifically designed for research purposes was established. Therefore, long‐term safety assessment was based on data derived from routine clinical practice. Third, the sample size is relatively small and derived from a single center, which may limit the generalizability of the findings.

Despite these limitations, the study also presents notable strengths. It reflects real‐world clinical practice, includes consecutive patients, and provides data over an extended timeframe, which is rarely available in the current literature on PN. Furthermore, the inclusion of cumulative treatment exposure and continued patient care within the same clinical setting enhances the reliability of safety observations.

In conclusion, PN‐HPT demonstrated a favorable safety profile in the periocular region in this real‐world cohort, with only mild and transient adverse events and no delayed complications identified in medical records over a 2‐year timeframe. Further prospective studies with larger sample sizes and standardized follow‐up protocols are warranted to confirm these findings and to better characterize the long‐term safety of polynucleotides in aesthetic medicine.

## 5 Conclusion

In this real‐world retrospective study, intradermal treatment with polynucleotides (PN‐HPT) in the periocular region demonstrated a favorable safety profile. Across 91 treatment sessions, only mild and self‐limited adverse events were observed, with no moderate or severe complications recorded.

Importantly, no delayed adverse events were identified in the medical records over a 2‐year timeframe, despite repeated treatments and increased cumulative exposure in a substantial proportion of patients. These findings support the tolerability of PN‐HPT in routine clinical practice, including with repeated use over time.

Although the retrospective design and relatively small sample size warrant cautious interpretation, this study provides clinically relevant real‐world evidence regarding the safety of polynucleotides, particularly in the periocular region, where safety considerations are critical.

Further prospective studies with larger cohorts and standardized follow‐up protocols are needed to confirm these findings and to better define the long‐term safety profile of polynucleotides in aesthetic medicine.

## Author Contributions

The author was responsible for the conception and design of the study, data collection, data analysis, interpretation of results, and manuscript preparation.

## Funding

The author has nothing to report.

## Ethics Statement

This study was conducted in accordance with the principles of the Declaration of Helsinki. Given its retrospective observational design based on anonymized medical records, formal ethical approval was not required according to local regulations. Informed consent was obtained from all patients for treatment. Due to the retrospective nature of the study and the use of anonymized data, additional consent for publication was not required.

## Conflicts of Interest

The author declares no conflicts of interest.

## Data Availability

Research data are not shared.
